# Development of and satisfaction with a short-term outpatient preventive medical service for working informal caregivers: a study protocol of a non-randomized prospective pilot study

**DOI:** 10.1186/s40814-026-01925-4

**Published:** 2026-09-15

**Authors:** Sophia Boesl, Anna Pendergrass, Elmar Graessel, Natascha Lauer, Annabèlle Mesloh, Elke Hüttenrauch, Antonia Keck, Johanna Schmidt

**Affiliations:** 1 Section of Medical Psychology and Medical Sociology, Department of Psychiatry and Psychotherapy, Uniklinikum Erlangen, Friedrich-Alexander-Universität Erlangen-Nürnberg (FAU), Erlangen, Germany; 2 Center for Health Services Research in Medicine, Department of Psychiatry and Psychotherapy, Uniklinikum Erlangen, Friedrich-Alexander-Universität Erlangen-Nürnberg (FAU), Erlangen, Germany; 3 Department for Dementia Research and Nursing Science, University for Continuing Education Krems, Dr.-Karl-Dorrek-Straße 30, Krems, 3500 Austria; 4 Tourismus Oberstdorf, Prinzregenten-Platz 1, Oberstdorf, 87561 Germany; 5 Oberstdorf, 87561 Germany

**Keywords:** Caregivers, Caregiver burden, Preventive health programs, Pilot study

## Abstract

**Background:**

About 80% of care receivers (CRs) in Germany are cared for at home by family members or friends. These informal caregivers (CGs) often report a high burden from caregiving, which negatively affects their mental and physical health and often results in reduced work hours. CGs with statutory health insurance can apply for preventive medical services. A change in the law has allowed the duration of service to be split. Recent research postulated specialized digital and analog multi-component interventions to protect CGs’ health, which the pAKur project wants to develop and implement into public health. This pilot will focus on the development of and satisfaction with an outpatient preventive medical service for CGs.

**Methods:**

The non-randomized, prospective pilot study will be performed on-site in Oberstdorf, Bavaria (Germany) and online from April 2026 to January 2027. Working CGs with a moderate CG burden caring for a CR with a moderate need for assistance will be included. The intervention will be developed in participation with CGs and experts in the corresponding field. Two groups (A and B) will participate in the same on-site treatment but will differ in the intensity of the digital support phase (A: low; B: high) and the location of the refresher treatment (A: on-site; B: digital). Within the framework of a mixed-methods design, quantitative and qualitative data will be collected: the main outcome will be CGs’ satisfaction with the outpatient preventive medical service, assessed with the Client Satisfaction Questionnaire and qualitative interviews. For secondary outcomes, several aspects of the CGs’ mental and physical health will be assessed via questionnaires and qualitative interviews. Quantitative data will be analyzed descriptively and explored with MANOVA; qualitative data will be examined with content analysis.

**Discussion:**

Potential issues regarding recruitment, organization, and dropout due to intervention duration will be addressed with financial incentives, support from the tourism board, and personal contact during the intervention. This pilot marks the first step in integrating the CGs’ needs into existing preventive medical services as well as testing components and structures for future interventions.

**Trial registration:**

German Clinical Trial Register (DRKS), ID: DRKS00039025; 01/22/2026

**Supplementary Information:**

The online version contains supplementary material available at https://doi.org/10.1186/s40814-026-01925-4.

## Background

In Germany, almost 6 million people were in need of long-term care in 2023, and 80% of them (ca. 5 million people) were cared for at home by informal caregivers (CGs) [64], i.e., family members or friends. CGs are often highly and chronically stressed by the caregiving situation. It often impairs their quality of life [3] and results in severe negative short- and long-term effects on their psychological and physical health [1, 18, 19, 62]. As a result, their general cognitive and physical performance capacity is also often impaired [54]. As caregiving requires physical and mental strength, the CG burden and its consequences affect not only the CG but also the caregiving situation and consequently the care receiver’s (CR’s) quality of life as well [1]. Working CGs are particularly at risk of psychosomatic or physical illnesses due to the double load of work and caregiving [22]. Thus, in order to be able to continue the caregiving, they often reduce their work hours or quit their jobs [59, 60]. Losing this income doubles down on the already existing financial burden from caregiving [2] and thus affects the country’s economy [67].

Support and relief services can help reduce the burden and enable CGs to continue working. Such services are available and partially covered for CRs with statutory health insurance and their CGs in Germany, depending on the CR’s care level. The care level (from 1 to 5) represents the need for assistance, with higher levels representing more need for assistance. The care level is determined by the *Medical Service of the German Health Insurance.* It is based on an assessment of the CR’s need for assistance in six areas of everyday life that are presented in the German Social Security Code (SGB XI §13). However, due to several barriers, e.g., lack of information, worries, availability, financial issues, or organizational issues [12, 55], CGs do not use support or relief services as much as they would like to [58]. Therefore, they often do not receive the help they need to cope with everyday life, thus risking their health. Maintaining CGs’ health with early prevention and sustainable relief strategies that reach CGs and can change their situation needs to be a societal goal. Preventive medical services provide an opportunity for CRs to preserve their health. In Germany, people with statutory health insurance can apply for outpatient or inpatient preventive medical services to prevent illness or mitigate their progression (SGB V §23,2) every 3 years. Outpatient treatment is preferred over inpatient treatment [24]. However, all other local treatment options need to be exhausted, and the patient’s physician needs to issue a recommendation for the preventive medical service. If the application is approved, patients in outpatient preventive medical services have access to 3 weeks of medical and therapeutic treatments at state-recognized health resorts [24]. Accommodations and food must be self-organized and are partly financially supported by the statutory health insurance. Since January 2024, it has been possible to split the time of the outpatient preventive medical services into two shorter stays, making it easier for working CGs to access the outpatient preventive medical services [32].

Current research has been calling for interventions to be implemented to reduce CG burden in public health for a broad spectrum of CGs for many years [2, 18, 49, 51, 70]. In light of the abovementioned changes in the law and the recommendations from previous research, we plan to design an outpatient preventive medical service for working CGs: the pAKur project (“*pA”* is an abbreviation for the German word for caregiver,“*Kur”* is the German word for a preventative medical service). It will use the already established structures of existing outpatient preventive medical services while including shorter treatment times and tailoring a multi-component treatment program to the needs of working CGs.

Studies have shown that multi-component interventions are more sustainable in reducing CG strain than single-component interventions [31, 49]. According to the literature [41, 62], the components should consist of psychotherapeutic strategies (i.e., psychoeducation or stress management), the provision of social support, the provision of care-related knowledge, and the implementation of physical exercises and relaxation. Additionally, providing information about and establishing contact to support the relief services can help increase the extent to which CGs use these services [55]. Offering these components in a group setting with other CGs as well as away from their home environment is particularly beneficial [31]. However, interventions that can be administered at home also seem to improve the CGs’ health [40, 73] and might reduce organizational effort for the CGs. In summary, it seems sensible to include both on-site and at-home as intervention settings to design the most effective and suitable outpatient preventive medical service possible.

It is the overall aim of the pAKur project to improve the CGs’ situation. To strengthen their mental and physical health and thus prevent illness, we plan to help CGs reduce their stress and strain while helping them improve their quality of life. Furthermore, we want to test shorter and in part digital versions.

The aims of our pilot study are to (1) develop an outpatient preventive medical service for and in cooperation with working CGs and (2) explore CGs’ satisfaction with the service. Additionally, we aim to (3) clarify the effect of the service on individual outcomes and potential differences that depend on group assignment and (4) identify the need, feasibility, and acceptance of the outpatient preventive medical service and its different variants.

## Methods/design

### Study design and setting

**Fig. 1:**
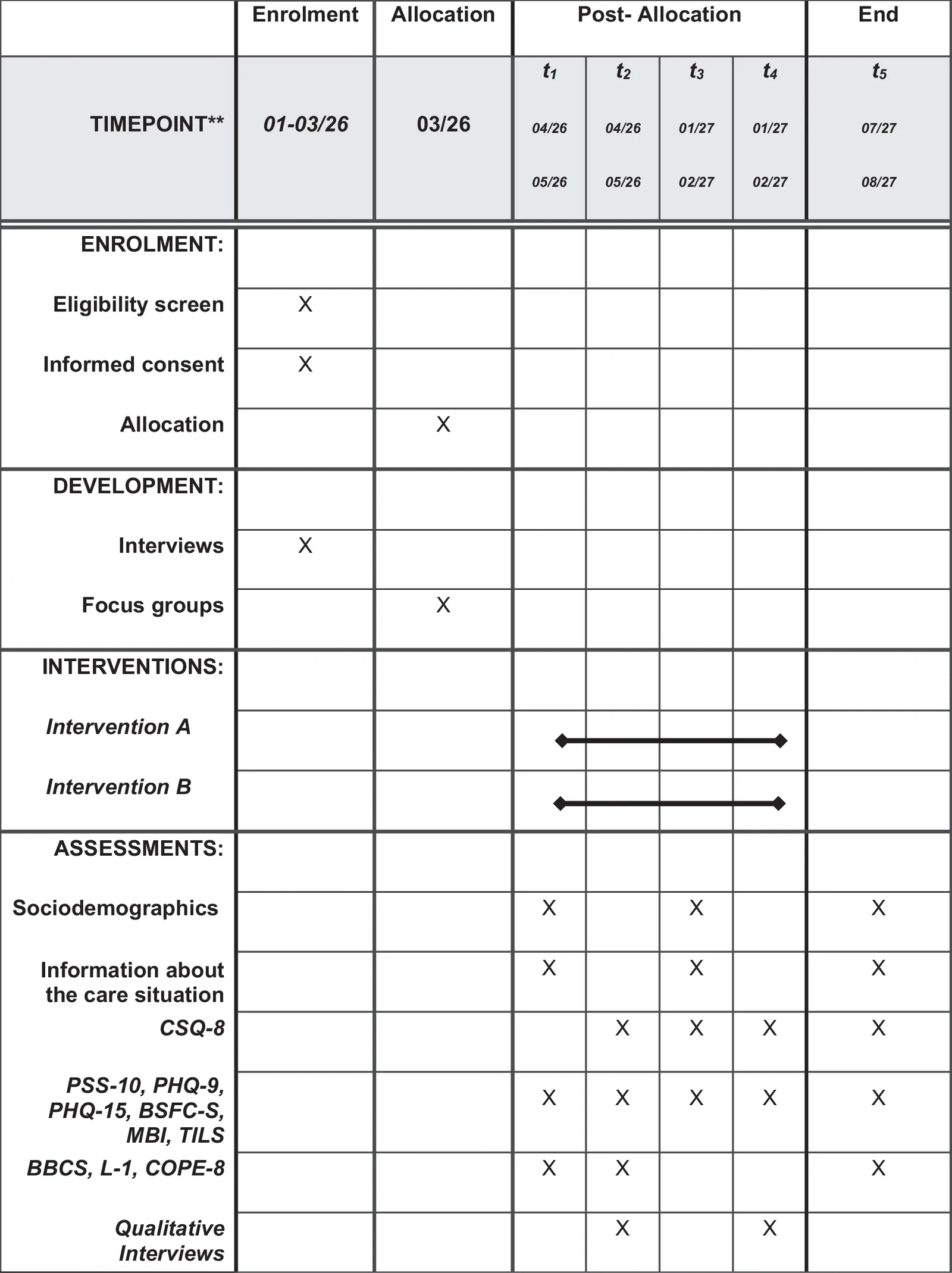
Timeline for enrollment, intervention, and measurements in accordance with the SPIRIT 2025 guidelines *Note:* CSQ-8: Client Satisfaction Questionnaire-8, PSS-10: Perceived Stress Scale-10, PHQ-9: Patient Health Questionnaire-9, PHQ-15: Patient Health Questionnaire-15, BSFC-S: Burden Scale for Family CGs- Short form, MBI: Maslach Burnout Inventory, TILS: Three-item Loneliness Scale, BBCS: Benefits of Being a CG Scale, L-1: General life satisfaction, COPE8: Coping Orientation to Problems Experienced 8 Questionnaire

To test the CGs’ satisfaction with the developed outpatient preventive medical service, we will conduct a non-randomized prospective intervention study with two arms and a follow-up. Allocation will be based on the CGs’ preferences. Written informed consent will be obtained from every participant before the on-site treatment begins. The pilot study is coordinated by the Centre for Health Services Research in Medicine in Erlangen, Bavaria, Germany. The on-site treatment program will be based in community facilities and a health resort facility (Klinik Hohes Licht) in Oberstdorf, Bavaria, Germany. On-site treatment will take place from April to May 2026 and January 2027. The digital treatment program will be hosted on Cisco’s WebEx video platform, which uses end-to-end encryption (E2EE) and is in accordance with the EU General Data Protection Regulation (GDPR). Consent to use Cisco’s WebEx will be part of the written declaration of consent that participants will sign at the beginning of the intervention. The intervention will last from April 2026 to January 2027. Figure 1 presents an overview of the timeline for enrollment, intervention, and measurements. The pilot study protocol is reported in line with the SPIRIT 2025 guidelines [15, 29]. The SPIRIT 2025 checklist can be found as an additional file (see Additional file 1). The pilot study was registered at Deutsches Register Klinische Studien (DRKS), ID: DRKS00039025,01/22/2026 (https://www.drks.de/search/de/trial/DRKS00039025/details).

### Participant characteristics

**Fig. 2:**
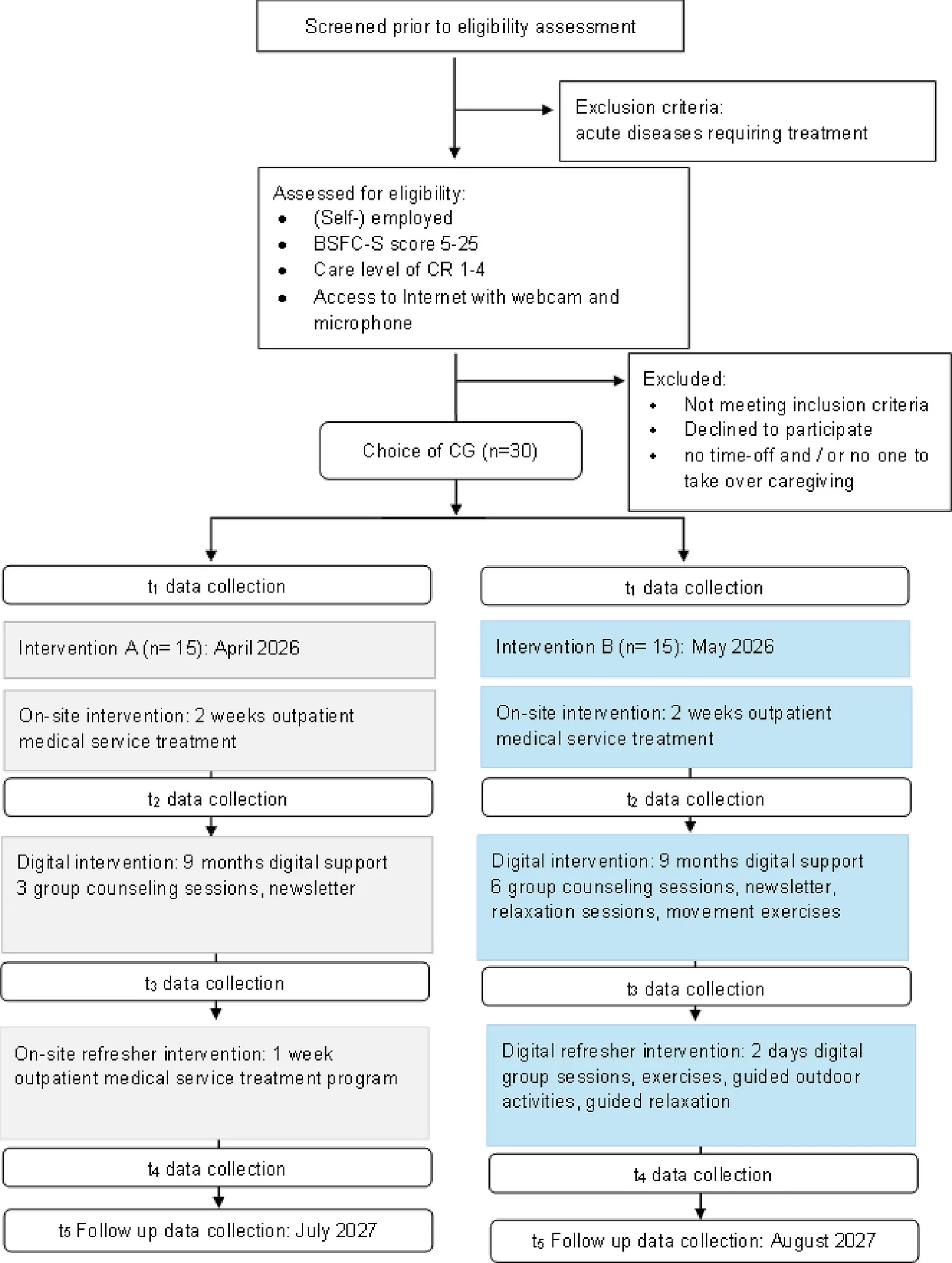
Participant flow *Note*: CG: caregiver; CR: care receiver; BSFC-S: Burden Scale for Family Caregivers-Short form

The following inclusion criteria needed to be met by CGs: being able to take part in the intervention by making arrangements for time off (e.g., with their employers) and for caregiving for their CR; being employed or self-employed; being moderately to highly strained by caregiving according to their score on the caregiver scale–short form ([25]; 5–25 points); being of legal age; caring for a CR with care levels 1–4; sufficient knowledge of German to be able to take part in conversations, interviews, and interventions; being able to use our digital services, e.g., by owning a PC, laptop, or smartphone with access to the internet and a webcam and microphone; giving informed consent to take part in the intervention. CGs with acute diseases that need treatment constraining participation will be excluded. For an overview of the participant flow, see Fig. 2.

### Intervention

#### Phase 1: development of the outpatient medical service

To develop the outpatient preventive medical service tailored to the working CGs’ needs, semi-structured qualitative interviews based on previous work were conducted in February 2026 with five German CGs who were recruited from a self-help online forum. The semi-structured interviews allow flexibility in conversations to improve comprehension and enable interviewers to ask follow-up questions [47]. The interview guide is organized so that it moves from general to specific [53]. Its themes are based on previous research [11, 63] and have been expanded and adapted to the present intervention. They consist of CGs’ needs and wishes regarding the preventive medical service, access barriers and facilitators, aims that can be reached through such treatment regarding behavioral changes, and conditions for a positive experience regarding travel and accommodations. Based on previous studies [63], the interviews will last between 30 and 50 min each. The main topics from the interviews were used as a basis for discussion in a focus group in February 2026 consisting of CGs, scientists, and experts in the field. The focus group comprised four sections on the topics of content, needs, feasibility, and acceptance. For each section, corresponding guiding questions were used.

#### Phase 2: execution of the outpatient medical service treatment

The outpatient preventive medical service will be split into three parts: a 2-week on-site outpatient treatment program in Oberstdorf, Bavaria, Germany in April and May 2026; a 9-month phase of digital support to implement the newly gained strategies and knowledge into everyday life; and a refresher treatment program in January 2027. To test whether more flexible and digital options could suit the CGs’ situation better, two different versions should be established with the same overall treatment hours but with differences regarding analogous treatment program time (group A: 2 weeks + 1 week, both on-site; group B: 2 weeks on-site + 2 days digital) and digital support (group A: 9 months of only a few sporadic occurrences of digital contact; group B: 9 months regular digital contact). To enable participants to plan time off work and arrange for caregiving for the CR, group allocation will be based on the CGs’ choice after they receive information about the schedule, dates, and procedures in both groups. Thus, no blinded group assignment can be provided. To detect possible confounders in group choice, participants will be asked about the reasons for choosing the respective group in qualitative interviews after the intervention.

The 2-week on-site treatment program will take place in rooms at the clinic (Klinik Hohes Licht), in community rooms, and outdoors in Oberstdorf, Bavaria, Germany during the day. Participants will spend the nights and weekends in accommodations organized by the local tourism board (Tourismus Oberstdorf). They will receive a financial allowance of 80€ per night as an incentive.

**Fig. 3:**
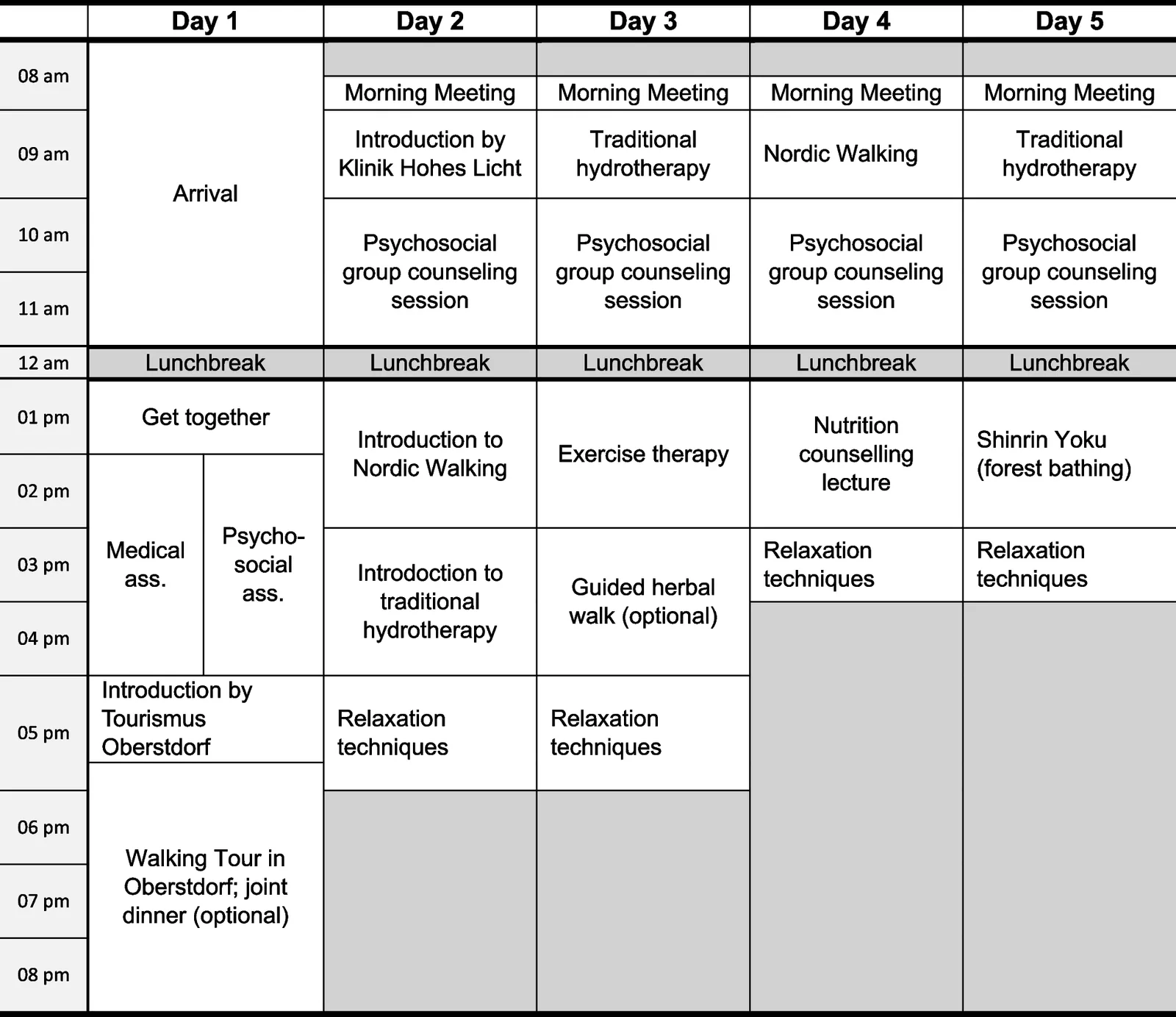
Example representation of the treatment plan for week 1 Note. Free time to spend as CGs wish in gray

Regarding the treatment program, overall results from the interviews and the focus group will be combined with already existing evidence-based intervention elements, i.e., psychoeducation [68], elements from Acceptance and Commitment Therapy (ACT; [75]), cognitive behavioral therapy [39], systemic therapy [71], mindfulness [23], and relaxation techniques, e.g., progressive muscle relaxation (PMR; [48]). All psychosocial elements of the program will be conducted by psychologists. Additionally, due to its location in a region with a healing climate, the treatment program will contain elements of forest bathing (Shinrin-Yoku) to improve the CGs’ mental health [34, 46, 50]. Activities such as movement therapy [65], Nordic Walking, traditional hydrotherapy [66], and nutritional counseling will be included and conducted by trained specialists. The daily morning meeting will allow participants to ask questions voice concerns. During these meetings, participants will be asked to disclose which activities they took part in the previous day and share their opinions on these acitvites. For an example treatment program plan, see Fig. 3.

Regarding the digital support after the on-site treatment program, each participant will, if they agree in advance, receive a monthly email newsletter that will serve as a reminder of already discussed content, skills, and methods. Participants of both groups will take part in group counseling sessions with psychologists (group A: 3 sessions; group B: 6 sessions over 9 months). The goal is to discuss participants’ issues with the planned changes while using solution-focused methods [17] and methods to resolve ambivalence from the Motivational Interviewing approach [45]. Participants of group B will also have access to digital face-to-face relaxation sessions, additional instructions about the methods they have already learned, and body and movement exercises. Participants will be asked to voice their opinions and record their attendance of the various acitivites via an online questionnaire.

For group A, the refresher week is planned as a 1-week on-site treatment program. It will be structured similarly to the first part of the intervention, including psychosocial group sessions, outdoor activities, as well as physical exercises and relaxation. The treatment program will take place in the clinic (Klinik Hohes Licht) and the community’s facilities as well as outdoors. Participants will stay in hotels, and 80€ financial support per night will be given as an incentive. For group B, participants will take part in a 2-day synchronous online treatment program at home. It will include psychosocial group sessions, physical exercises, and relaxation. In the end, all participants should have the same number of treatment program hours.

### Data collection and measures

#### Data collection and management

An intensive recruitment phase is carried out. Information about the project is made available to CGs through online forums, support groups, and care counseling services. In addition, CGs are informed about the project through articles in free health magazines and newspapers, as well as through other public outreach efforts (e.g., podcasts).

Quantitative and qualitative data will be collected throughout the intervention. Before every round of data collection, a trained student will send the questionnaires to the participants by mail. Afterwards, the trained students will call the participants and enter the participants’ pseudonymized answers into the REDCap web platform [27] hosted at Uniklinikum Erlangen. This procedure will enable a complete data set by reducing missing answers. Data will be collected between April 2026 and August 2027.

All identifying information will be encrypted, and participants will be assigned an ID number. Information that is needed to register for accommodations will be saved in a different password-protected document. All data will be saved on local servers. The qualitative interviews will be conducted and recorded via the end-to-end encrypted videoplatform WebEx by Cisco and transcribed by trained students. WebEx by Cisco is hosted on servers in the European Union. The interview will only be recorded with the written informed consent of the interviewees. Recordings will be used exclusively for scientific evaluation and will be deleted after they have been transcribed.

#### Sociodemographics and information about the caregiving situation

Regarding the caregiving situation and information about the CR, potential changes in the following information will be explored at baseline (*t*_1_), before the refresher intervention (*t*_3_), and at follow-up (*t*_5_):

- the CRs’ *care level*, as evaluated or reevaluated by the Medical Service of the German Health Insurance. Higher care levels indicate a greater need for long-term care and assistance in everyday life ranging from 1 (minor impact on independence or abilities) to 5 (significant loss of independence or abilities, along with the need for extensive nursing assistance), as defined by the German Social Code XI (SGB XI).
- the *daily informal care time*, e.g., average number of hours (range 0–17 h) the CG spends providing services to the CR for support in Activities of Daily Living (ADLs), Instrumental Activities of Daily Living (IADLs), and supervision (based on the Resource Utilization in Dementia questionnaire) [74], estimated by the CGs.
- the *CGs*’ *perceptions* of the caregiving situation, assessed with two items (“How do you rate your current caregiving situation in general?" and “How do you currently rate your ability to cope with the caregiving?”) on a scale ranging from 1 (negative/I am not succeeding at all) to 10 (positive/I am succeeding completely).
- the current *subjective relationship quality* between the CG and the CR, assessed with the question “How would you rate the quality of the relationship between you and the person you support or care for?” [5], which can be answered “negative,” “neutral,” or “positive.” To avoid socially desirable response bias [10], negative and neutral answers will be combined.
- Information on the *use of formal support services* in the last 3 months, based on the Resource Utilization in Dementia questionnaire [74].
- *Sociodemographics*, including information about the CGs’ employment status and monthly income.
- the CGs’ *pre-existing conditions* as well as *medication intake*.

Additional sociodemographic data (sex of the CG and CR, age of the CG and CR, migration background of the CG and CR, education in years of the CG, duration of informal caregiving, reason for caregiving) will be assessed at baseline (*t*_1_).

#### Primary outcome measure

We will use the participants’ overall satisfaction with the outpatient preventive medical service as the primary outcome measure after each part of the intervention and at the follow-up (*t*_2_, *t*_3_, *t*_4_, *t*_5_). Satisfaction will be measured quantitatively using the German version of the Client Satisfaction Questionnaire [4], an economical, valid, and reliable scale [35, 61] to depict the overall level of patient satisfaction with healthcare services. Participants will answer 8 items on a 4-point Likert scale. Sum scores will range from 8 to 32, with higher scores indicating higher satisfaction. A sum score of 23 or higher indicates satisfaction with the program [35]. Additionally, satisfaction will be assessed via qualitative interviews at *t*_2_ and *t*_4_. For more information regarding the qualitative interviews, see the Qualitative Data section.

Participants will rate their satisfaction with the individual activities they attended both on-site and online in form of feedback-oriented visual analog scales [21].

#### Secondary outcome measures

For secondary outcomes, we will assess sum scores at baseline, after each part of the intervention, and at follow-up (*t*_1_, *t*_2_, *t*_3_, *t*_4_, *t*_5_), and we will explore the change in sum scores for the following instruments:

- *Perceived Stress Scale-10* [16], German version [33]. This instrument includes the scales helplessness (6 items, score 6–30), self-efficacy (4 items, score 4–20), and overall perceived stress on a 5-point Likert scale ranging from 1 (never) to 5 (very often). The total sum score ranges from 10 to 50, with higher scores indicating more perceived stress.
- *Patient Health Questionnaire-9* (PHQ-9; [36]), German version [26]. This instrument measures depression severity with 9 items rated on a 4-point Likert scale ranging from 0 (not at all) to 3 (nearly every day). The sum score ranges from 0 to 27, with higher scores indicating more severe depressive symptoms [36].
- *Patient Health Questionnaire-15* (PHQ-15; [37]), German version [26]. This instrument captures psychosomatic complaints with 15 items rated on a 3-point Likert scale ranging from 0 (not bothered at all) to 2 (bothered a lot). The sum score ranges from 0 to 30, with higher scores indicating more severe symptoms [37].
- *Burden Scale for Family CGs-short form* (BSFC-s; [25]), German version. This instrument captures CGs’ subjective burden with 10 items rated on a 4-point Likert scale ranging from 0 (strongly disagree) to 3 (strongly agree). The sum score ranges from 0 to 30 with higher scores indicating higher subjective burden.
- *Maslach Burnout Inventory* (MBI; [42]), German version [13]. This instrument contains 22 items rated on a 7-point Likert scale ranging from 0 (never) to 6 (every day). It captures Emotional Exhaustion (9 items, with the sum score ranging from 0 to 63 such that higher scores indicate greater feelings of exhaustion), Depersonalization (5 items, with the sum score ranging from 0 to 45, such that higher scores indicate greater feelings of depersonalization), and Personal Accomplishment (8 items, with the sum score ranging from 0 to 72, such that higher scores indicate greater feelings of competence).
- *Three-item Loneliness Scale* (TILS; [30]), German version. This instrument measures participants’ loneliness with 3 items rated on a 3-point Likert scale ranging from 1 (hardly ever) to 3 (often). The sum score ranges from 1 to 9. Scores ranging from 3 to 5 indicate no loneliness, whereas scores ranging from 6 to 9 indicate loneliness.

Additionally, the changes in sum scores at baseline, after the first on-site intervention, and at follow-up (*t*_1_, *t*_2_, *t*_5_, respectively) for the following instruments will be used as secondary outcomes:

- *Benefits of Being a CG Scale* (BBCS; [52]), German version. This instrument captures the CGs’ benefits from informal caregiving with 15 items rated on a 4-point Likert scale ranging from 0 (strongly disagree) to 4 (strongly agree). The sum score ranges from 0 to 60, with higher scores indicating more benefits from caregiving.
- *General satisfaction with life,* German instrument (Allgemeine Lebenszufriedenheit (L-1; [6]). This one-item scale is rated on an 11-point scale ranging from 1 (not satisfied at all) to 11 (completely satisfied).
- *Coping Orientation to Problems Experienced 8* Questionnaire (COPE-8; [14]), adapted and translated into German. This instrument measures functional (emotion- and problem-focused coping) and dysfunctional coping strategies. Higher scores indicate more frequent use of the corresponding coping strategies.

#### Qualitative data

Qualitative data will be assessed with respect to the feasibility, need, and acceptance of the on-site intervention and the digital support intervention (*t*_2_, *t*_4_) with semi-structured qualitative interviews. The structure and themes will be based on previous research [63] and adapted to the current pilot study. Participants will also be asked for their reasons for choosing their respective group. The qualitative interviews will be conducted and recorded via WebEx by Cisco and transcribed verbatim by trained students. Recordings will be used exclusively for scientific evaluation and will be deleted after they are transcribed.

### Statistical analysis

We plan to form two groups of 15 participants each. This compromises between the recommendation for both sample sizes for pilot studies [28] as well as group sizes for group interventions in RCTs [9]. Data will be analyzed within the framework of a mixed-methods approach with a parallel design with the IBM Statistical Package for the Social Sciences (SPSS) software, version 29. Results from the quantitative and qualitative data analyses will be compared accordingly. Quantitative and qualitative findings will be integrated at the interpretation stage.

#### Descriptive statistics

To describe the study population, means or medians and standard deviations will be calculated for metric variables. Frequencies and proportions will be calculated for categorical variables.

#### Inferential statistics

Multivariate analyses (e.g., mixed ANOVAs) will be applied to estimate the effect size of the planned primary outcome for the RCT (BSFC-s). This estimate will inform the case numbers calculation for the following RCT, while accounting for an overestimation due to the small sample size [38]. Additionally, pre–post changes, group effects and group-by-time interactions will be explored using effect sizes and confidence intervals. A responder analysis will be conducted to determine which CGs benefit the most from the interventions.

#### Qualitative data

Qualitative data will be analyzed with the qualitative content analysis approach [43].

#### Progression criteria

Thresholds for feasibility (recruitment rate, retention rate, and adherence rate) and satisfaction, informing the decision to proceed to an RCT (“Go”) or otherwise to amend the study design [44] were established (see Table 1). Threshold values for recruitment rate [20], retention rate [56, 57], adherence rate [57, 72], and satisfaction [57] were derived from comparable projects addressing the same target population of CGs. Thresholds were set conservatively to account for the anticipated recruitment challenges [9], the duration of the project, and the high probability of change in the informal care arrangement over the course of the study, which would result in participant exclusion.

**Table 1:** Progression criteria with benchmarks

Domain	Metric	Go
Recruitment rate	Proportion of eligible CGs consenting to participate	≥ 65%
Retention rate	Proportion of CGs completing the *t*_5_ follow-up data collection	≥ 60%
Adherence rate	Proportion of sessions attended (on-site and digital)	≥ 80%
Satisfaction	Proportion of CGs with a sum score ≥ 23 on the primary outcome measure (ZUF-8), averaged across all data collection time points	≥ 75%

*Note*: *CG* caregiver, *ZUF-8* German version of the Client Satisfaction Questionnaire

## Discussion

With our pAKur project, we are putting into practice the call for interventions and the implementation of research findings on the burden of CGs. We aim to reduce their burden and increase their mental and physical stability. With the knowledge of CGs and experts in the field, we want to develop an outpatient preventive medical service for working CGs and test the CGs’ satisfaction with the service. Additionally, we will determine how to organize the recruitment and the intervention during the pilot, and we will use these organizing principles in the upcoming RCT as well.

Determining the best approach for recruitment will be especially important, as the main issue of the project is expected to be the recruitment of the participants. In general, many CGs do not use support services. CGs are often unaware of their caregiver burden and their own needs or they do not prioritize them [12]. Thus, we expect an initially small number of interested CGs, as they may not recognize their need for a preventive medical service. It is necessary to raise awareness of the impact of caregiver burden on the physical and psychological well-being of CGs, while also providing information about the project in relevant journals, newspapers, and newsletters for CGs [7]. Targeted recruitment of participants through clear project descriptions, as well as the involvement of CGs who support the project or have already participated, may also facilitate recruitment [20].

Due to the short recruitment time, we expect that many CGs will not be able to participate because there is no one to take over for them in providing informal caregiving for their CR or because they lack vacation days [68]. In this case, we plan to postpone one intervention group from April to November to give the CGs more advance notice to help them plan. Additionally, we plan to cooperate with major employers, who can grant special leave for their employed CGs. We have also ensured that the refresher week will take place in 2027 so that CGs can spread the required vacation days over 2 years.

In line with outpatient preventive medical service from the statutory health insurance [24], CGs will need to cover some of the costs of the treatment program by themselves, as some costs are not covered (e.g., accommodations, travel fees). This issue might reduce the attractiveness of the program and might also exclude CGs who cannot afford such costs. With the 80€ financial support per night, CGs already need to cover fewer expenses in our treatment program than in the regular outpatient preventive medical service. However, we will raise the daily financial support from 80€ to 100€ per participant. By doing so, we hope to further increase the attractiveness of our treatment program and to enable CGs with different social backgrounds to attend [68].

We expect some dropout due to the length of the intervention (approximately 9 months). The informal caregiving arrangement could change (e.g., if the CR moves into a nursing home or passes away), in which case the CGs will not be allowed to participate, as their data could not be used from that point forward [69]. However, the length of the intervention will allow us to support CGs for a longer period of time as postulated by the literature [31]. As part of the digital support, we can encourage and help implement the changes in behavior into the CGs’ everyday life [8]. We also hope to prevent potential dropout due to motivational issues by providing regular personal contact.

A greater organizational effort is expected regarding the availability, booking, and potential cancellation of hotels in a popular tourist area such as Oberstdorf in Bavaria, Germany. Many preventive treatment programs from the statutory health insurance are embedded in clinics. However, most of these clinics are often fully booked months in advance. By outsourcing the accommodations and some of the activities to facilities in the village, we hope to separate the availability of treatment from the resources in the respective clinic and thus enable more CGs to access preventive treatment programs. Additionally, to support the participants during the booking process, we will provide booking support via our partner from the local tourism board (Tourismus Oberstdorf). Thus, participants will be offered a project-specific landing page where booking is simplified by offering easy access to the cooperating accommodations.

While this pilot has some anticipated difficulties, it also has strengths. First, due to the participatory nature of the development of the outpatient preventive medical service, we can respond specifically to the CGs’ needs. Thus, we can develop a treatment program that is both helpful and realistic. Second, as data will be collected via phone, we expect little missing data. Third, by collecting data over a period of 9 months, we can examine the long-term development of the CGs. Fourth, due to the mixed-methods approach, we expect a comprehensive data collection that allows quantitative results to be explained and expanded by qualitative data. Fifth, with the overall results, we can further adapt the program to the participating CGs’ perspectives and experiences.

The information and knowledge gained from the pilot study, which will focus primarily on feasibility and satisfaction, will be used to plan an RCT that will focus on the sustainable implementation of the developed outpatient preventive medical service.

## Supplementary Information

Additional file 1. SPIRIT 2025 checklist of items to address in a randomized trial protocol.

## Data Availability

No datasets were generated or analysed during the current study.

## Abbreviations

CG: Informal caregiver
CR: Care receiver
SGB: German Social Security Code
pAKur: Outpatient preventive medical service for working caregivers
